# Supplementary material for: One-dimensionally oriented self-assembly of ordered mesoporous nanofibers featuring tailorable mesophases via kinetic control
Source: Nat Commun. 2023 Dec 9;14:8148. doi: 10.1038/s41467-023-43963-z (PMC10710411; doi:10.1038/s41467-023-43963-z)
Supplement: Supplementary file 1 — Supplementary Information [file 41467_2023_43963_MOESM1_ESM.pdf]

Supplementary Information

One-dimensionally oriented self-assembly of ordered mesoporous nanofibers featuring tailorable mesophases via kinetic control

Liang Peng[1,2†], Huarong Peng[1,3†], Steven Wang[2], Xingjin Li[1], Jiaying Mo[2], Xiong Wang[2], Yun Tang[1], Renchao Che[1], Zuankai Wang[2,4]*, Wei Li[1]*, and Dongyuan Zhao[1]*

[1]Department of Chemistry, Laboratory of Advanced Materials, Shanghai Key Laboratory of Molecular Catalysis and Innovative Materials, *i*ChEM and State Key Laboratory of Molecular Engineering of Polymers, Fudan University, Shanghai 200433, P. R. China

[2]Department of Mechanical Engineering and Research Center for Nature-Inspired Engineering, City University of Hong Kong, Hong Kong, 999077, P. R. China

[3]Department of Chemistry, The University of Hong Kong, Hong Kong, 999077, P. R. China

[4]Department of Mechanical Engineering, The Hong Kong Polytechnic University, Hong Kong 999077, P. R. China

[†]These authors contributed equally to this work

*To whom correspondence should be addressed.

E-mail: dyzhao@fudan.edu.cn (D. Z.);

weilichem@fudan.edu.cn (W. L.);

zk.wang@polyu.edu.hk (Z. W.)

Contents

1. Supplementary Methods

   1.1 Materials

   1.2 Synthesis process

   1.3 Characterization

   1.4 Application measurements

2. Supplementary Figures

   2.1 Formation process of the OMCFs (Fig. 1)

   2.2 Characterization of the OMCFs (Fig. 2-6)

   2.3 Control experiments (Fig. 7-14, Tab. 1)

   2.4 Mechanism exploration (Fig. 15-17)

   2.5 Electrochemical application of OMCFs (Fig. 18-22, Tab. 2)

   2.6 Water purification application of OMCFs cryogel (Fig. 23-27, Tab. 3)

3. Supplementary References

1. Supplementary Methods

1.1 Materials. Triblock copolymer Pluronic F127 ($EO_{106}PO_{70}EO_{106}$, M_w = 12600) was purchased from Sigma-Aldrich Corp. Phenol (C_6H_6O), hexamethylenetetramine ($C_6H_{12}N_4$, HMT), formaldehyde solution (CH_2O, 37 wt %), sodium hydroxide (NaOH), ammonia ($NH_3 \cdot H_2O$), Rhodamine B, Fast green FCF and ethanol (C_2H_6O) were purchased from Shanghai Chemical Corp. Pure water was deionized with a Milli-Q water purification system (Millipore). All chemicals were used directly without further purification.

1.2 Synthesis process

Preparation of resol precursor. The resol precursor (M_w = 500 - 5000) was prepared according to the previously reported method.[1, 2] In a typical procedure, 0.61 g of phenol was melted at 40 °C in a 100 mL flask and then mixed with 0.13 g of 20 wt% NaOH aqueous solution at 300 rpm. After 10 min, Formalin (37 wt%, 1.05 g) containing formaldehyde (13.0 mmol) was added dropwise, and the reaction mixture was stirred at 70 °C. Upon further stirring for 1 h, the mixture was cooled to room temperature and the pH value was adjusted to about ~ 7.0 by using HCl solution (0.6 M). After water was removed by vacuum evaporation below 50 °C, the final product was redissolved in ethanol (20 wt % ethanolic solution) for further use. The density of resol ethanolic solution is about ~0.8 g mL^{-1}.

Preparation of the ordered mesoporous carbon nanofibers (OMCFs). The ordered mesoporous carbon nanofibers were fabricated by a kinetically driven monomicelle oriented self-assembly approach using Pluronic F127 as the structure-directing agent,

HMT as the curing agent, phenolic resol as the carbon source. The synthesis route involved three steps: (i) the preparation of F127/Resol monomicelles, (ii) the subsequent self-assembly of the composite monomicelles into mesostructured polymeric nanofibers, and (iii) conversation of the polymeric nanofibers into OMCFs. Typically, in the first step, 1.0 mL of phenolic resol solution, 0.24 g of Pluronic F127, and 0.08 g of HMT were continuously added into a 200 mL flask with 80 mL of deionized water under stirring rate of 300 rpm. The stirring bar was 2 cm in length. After continuously stirring for 2 h at ambient temperature, the mixture was transferred into a 100 mL Teflon-lined stainless-steel autoclave and heated at 100 °C for other 24 h. After cooling down to room temperature, the well-developed mesoporous polymeric nanofibers were separated by centrifugation at 5900 g, followed by washing with deionized water for several times and freeze drying. Finally, the polymeric composites were pre-heated at 350 °C for 3 h and further kept at 800 °C for 2 h with a heating rate of 1 °C min^{-1} in N_2 atmosphere, resulting in the ordered mesoporous carbon nanofibers.

Synthesis of 3D OMCFs cryogel.

The hierarchical OMCFs cryogels were prepared through an ice-templating method. In a typical synthesis, 1.0 g of ordered mesoporous polymeric nanofibers (OMPFs) was dispersed in 300 mL of deionized water by stirring at 1000 rpm for 30 min, yielding uniform nanofiber dispersion with a concentration of 3.4 mg mL^{-1}. Then, 15 mL above mixing dispersion was transferred to a designed cylindrical Polytetrafluoroethylene (PTFE) mold (2.5 cm in diameter, 3.5 cm in depth), where the bottom was covered by

a copper gasket (0.2 cm in thick). Next, the whole mold was immersed in liquid nitrogen to generate a temperature gradient from bottom to top. After the dispersion was completely frozen, the device was placed in a freeze-dryer more than 24 h under 1.0 Pa pressure and -80 °C to generate the OMPFs cryogels. Finally, the OMPF cryogels were pre-heated at 350 °C for 3 h and further kept at 800 °C for 2 h with a heating rate of 1 °C min^{-1} in N_2 atmosphere to obtain the OMCFs cryogels.

The randomly linked OMCFs cryogels were prepared by the similar synthetic procedure except that the aqueous polymeric nanofiber dispersion was poured in a plastic pipe and followed by freezing in a refrigerator (-20 °C) for 24 h.

Control experiments

Change in the catalyst. The synthetic processes were performed similarly except that the catalyst was changed from HMT, to $NH_3 \cdot H_2O$, and to NaOH.

Change in the temperature. The synthetic processes were performed similarly except that the temperature was changed from 80, to 100, to 120, and to 140 °C.

Change in the HMT concentration. The synthetic processes were carried out similarly except that the HMT amount was changed from 0.2, 0.4, 0.8, to 1.6 g L^{-1}.

Change in the F127/Resol mass ratio. The synthetic processes were conducted similarly except that the F127/Resol mass ratio was changed from 0, 0.1, 0.3, to 0.6.

1.3 Characterization. Small-angle X-ray scattering (SAXS) measurements were carried out at a Nanostar U SAXS system (Bruker, Germany) using Cu-Kα radiation (40 kV, 35 mA). The wide-angle X-ray diffraction (XRD) pattern was conducted on a

Bruker D4 X-ray diffractometer (40 kV, 40 mA). Scanning electron microscopy (SEM) images were collected by a Hitachi S-4800 microscope (Japan) operated at 5 kV. Transmission electron microscopy (TEM) images were obtained using a JEM-2100 F microscope (Japan) operated at 200 kV. X-ray photoelectron spectroscopy (XPS) was performed on an AXIS ULTRA DLD XPS system (Shimadzu Corp, U.K.) using a monochrome Al X-ray source. N_2 adsorption-desorption isotherms were measured at a Micromeritics Tristar 3020 analyzer (U.S.A.) at 77 K. Before measurements, all samples were degassed under vacuum at 180 °C for 18 h. The specific surface areas were calculated by using Brunauer-Emmett-Teller (BET) method from the adsorption data at P/P_0 = 0.05 - 0.25. The pore size distributions (PSD) were estimated by using Barrett-Joyner-Halenda (BJH) model. The total pore volumes (V_{total}) were calculated from the adsorption branches at P/P_0 = 0.995. Raman spectrum was obtained by a Dilor LabRam-1B microscopic Raman spectrum (France) with a standard Ar laser. Thermogravimetric analysis (TGA) curve was performed on a Mettler Toledo TGA-SDTA851 analyzer (Switzerland) from 25 to 800 °C in an N_2 flow of 20 mV min^{-1} at a heating rate of 10°C min^{-1}. The UV-Vis spectra were recorded on a PerkinElmer Lambda 1050+ spectrometer. Infrared images were collected using a FLIR T1050sc infrared camera.

1.4 Application measurements

Electrochemical measurements. All electrochemical tests were performed using CR2032-type coin cells assembled in an argon-filled glove box. The working electrodes

were prepared by thoroughly mixing the mesoporous carbon nanofibers (80 wt%), conductive Super-P (10 wt%) and polyvinylidene fluoride (PVDF, 10 wt%) in N-methyl-pyrrolidone to form a slurry. Then, the slurry was uniformly coated onto copper (Cu) foil and dried at 100 °C for 12 h to obtain the working electrodes. The cells were assembled using sodium foil as the counter electrode, glass fiber as the separator and an electrolyte consisted of 1.0 M $NaPF_6$ in ethylene carbonate (EC) and dimethylcarbonate (DMC) (1 : 1 by volume). Galvanostatic measurements were carried out using a LAND CT2001A battery testing system (Wuhan LAND Electronic Co., Ltd) in the voltage range of 0.01 - 3.0 V. Cyclic voltammogram (CV) measurements were conducted on the CHI 660E electrochemical workstation (Shanghai Chenhua instrument Co., Ltd) at 0.2 mV s^{-1}. Furthermore, the sodium ion full cells were assembled by using mesoporous carbon nanofibers as an anode material and $Na_3V_2(PO_4)_2F_3$ as a cathode material, respectively. The mass ratio of mesoporous carbon nanofibers and $Na_3V_2(PO_4)_2F_3$ was controlled at 1 : 4 to balance the capacity. The electrochemical tests were carried out in the voltage range of 1.5 - 4.3 V.

Water purification measurements. For the dye separation experiment, two different organic dyes, namely Rhodamine B and Fast green FCF, were selected as model representative pollutants to get insight into the molecular filtration. The concentrations of each organic dyes were 40 mg L^{-1}. Next, the dye solutions were injected into the nanofilter device based on the OMCFs cryogel. The separation efficiency (η) of filtration process can be calculated by the following equation:

$$\eta\% = \frac{C_i - C_f}{C_i} \times 100$$

where C_i and C_f represent the organic dye concentrations before and after filtration process, respectively. For solar steam generation experiment, the OMCFs cryogel (radius 3 cm, heigh 1 cm) was put on the interface of air/water of beaker under a solar simulator (PLS-SXE300C). The evaporation rate was collected for 60 mins in a stable state, and the weight loss was recorded using an electronic mass balance (LICHEN FA2204). The surface temperature was recorded using an infrared camera (FLIR T1050sc).

2. Supplementary Figures

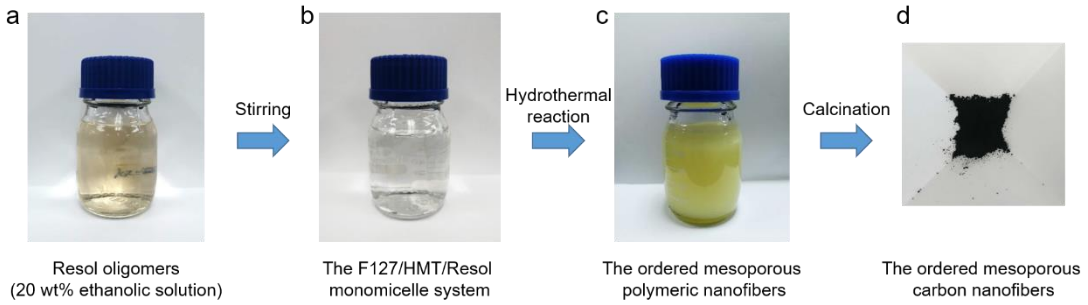

Supplementary Fig. 1. **Optical photographs of the formation process**. The ordered mesoporous carbon nanofibers prepared by the kinetically driven monomicelle oriented self-assembly approach, and followed by carbonization at 800 °C for 2 h in N_2 atmosphere.

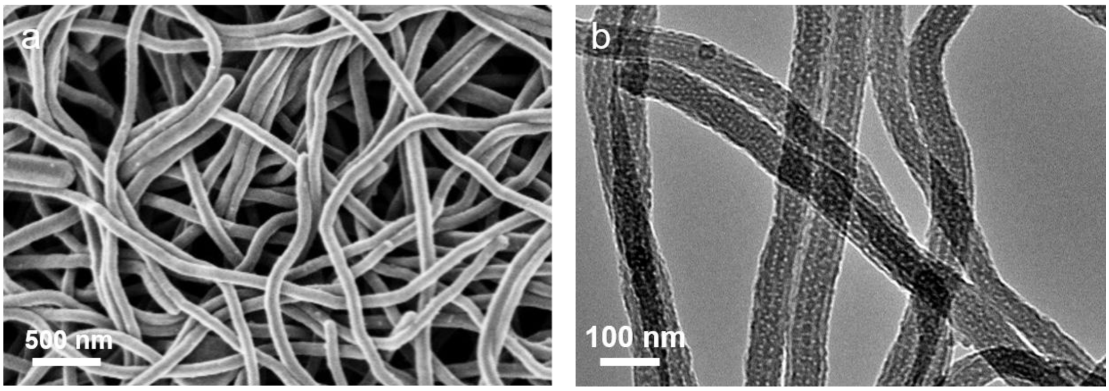

Supplementary Fig. 2. **Morphological and structural characterization of the ordered mesoporous polymeric nanofibers**. (a) SEM and (b) TEM images of the ordered mesoporous polymeric nanofibers prepared by the kinetically driven monomicelle oriented self-assembly approach.

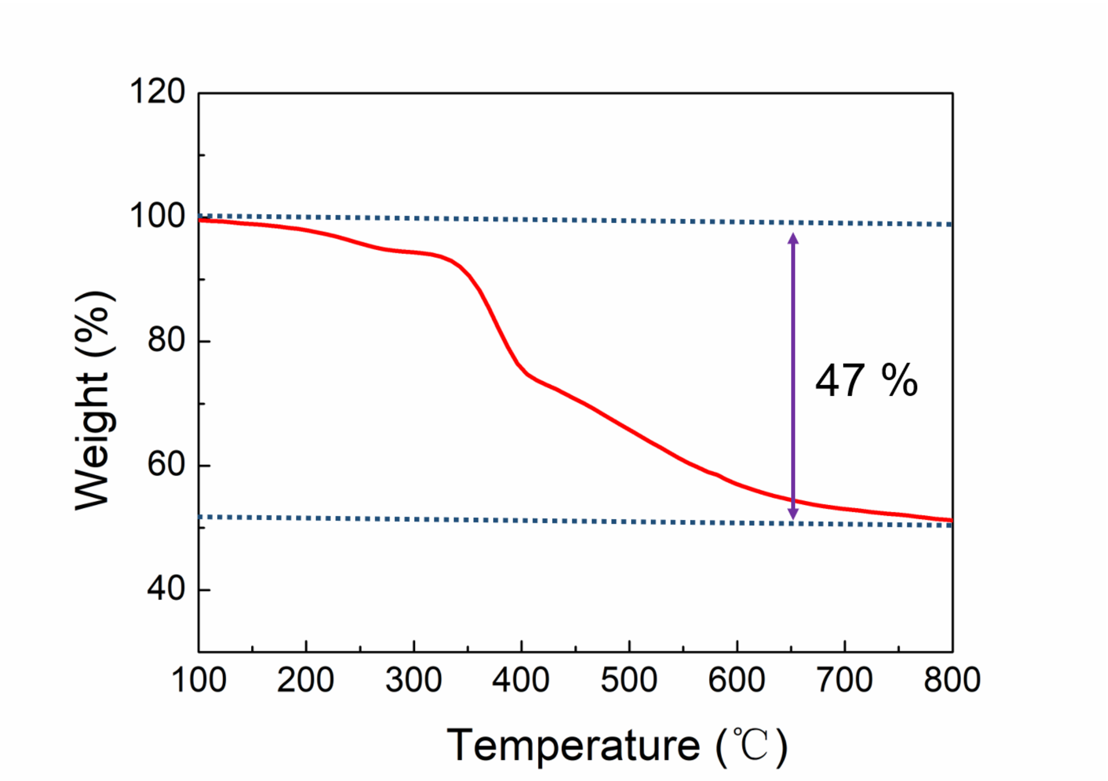

Supplementary Fig. 3. **The TGA of the ordered mesoporous polymeric nanofibers.** The ordered mesoporous polymeric nanofibers prepared by the kinetically driven monomicelle oriented self-assembly approach.

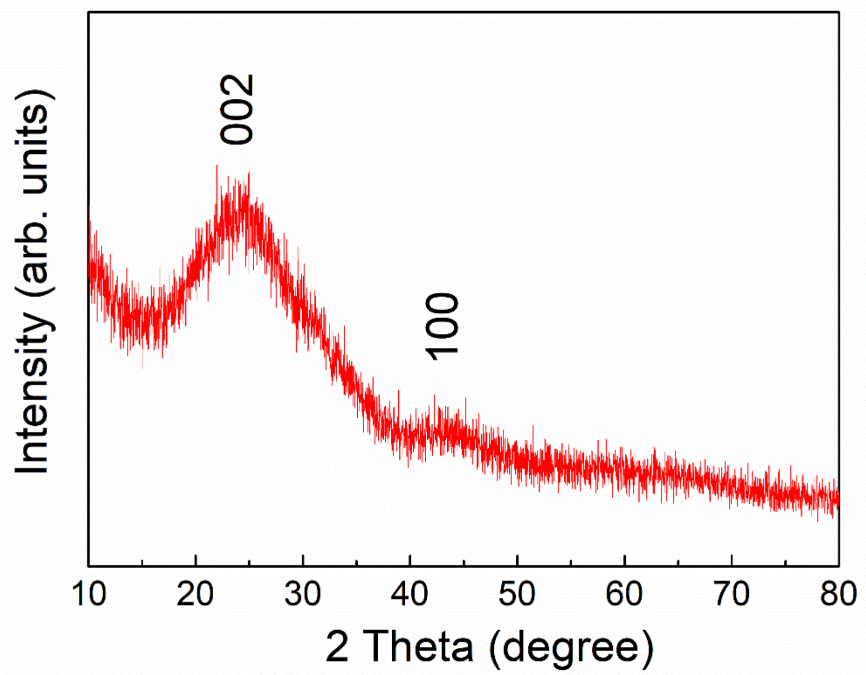

Supplementary Fig. 4. The XRD pattern of the ordered mesoporous carbon nanofibers. The ordered mesoporous carbon nanofibers prepared by the kinetically driven monomicelle oriented self-assembly approach after carbonization at 800 °C for 2 h in N_2 atmosphere.

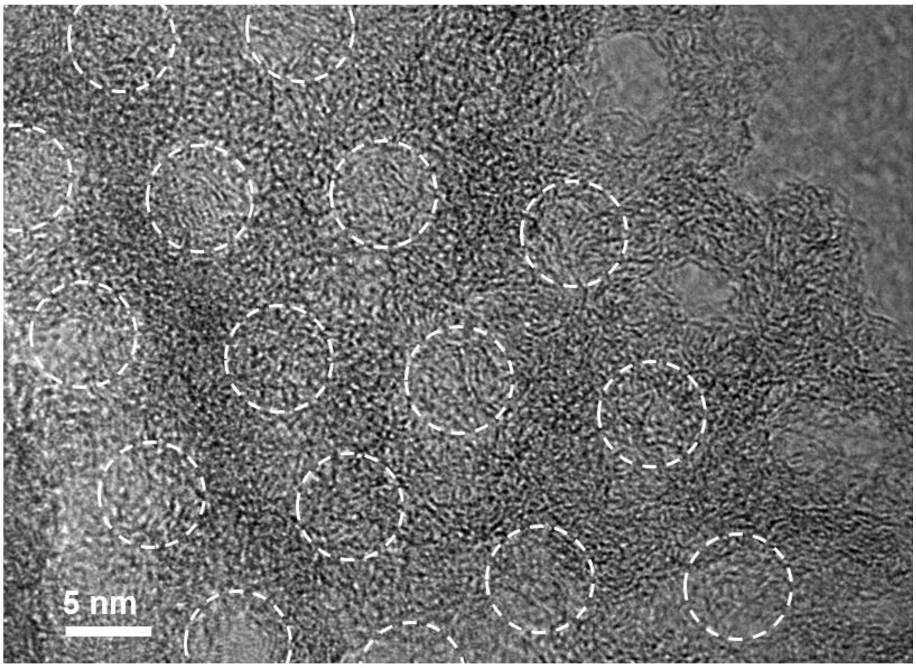

Supplementary Fig. 5. **The high-resolution TEM image of the ordered mesoporous carbon nanofibers.** The ordered mesoporous carbon nanofibers prepared by the kinetically driven monomicelle oriented self-assembly approach after carbonization at 800 °C for 2 h in N_2 atmosphere.

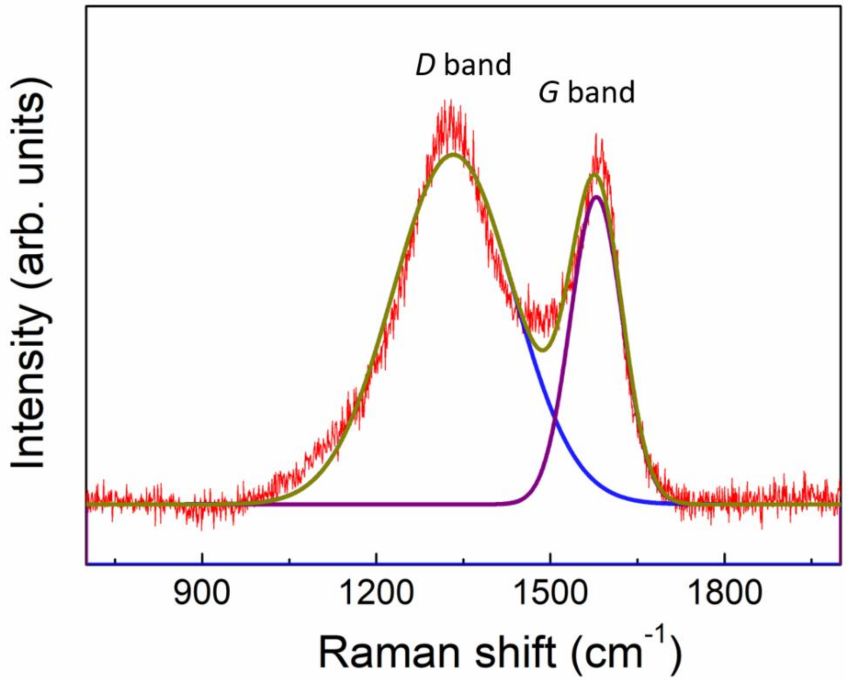

Supplementary Fig. 6. **The Raman spectrum of the ordered mesoporous carbon nanofibers**. The ordered mesoporous carbon nanofibers prepared by the kinetically driven monomicelle oriented self-assembly approach after carbonization at 800 °C for 2 h in N_2 atmosphere.

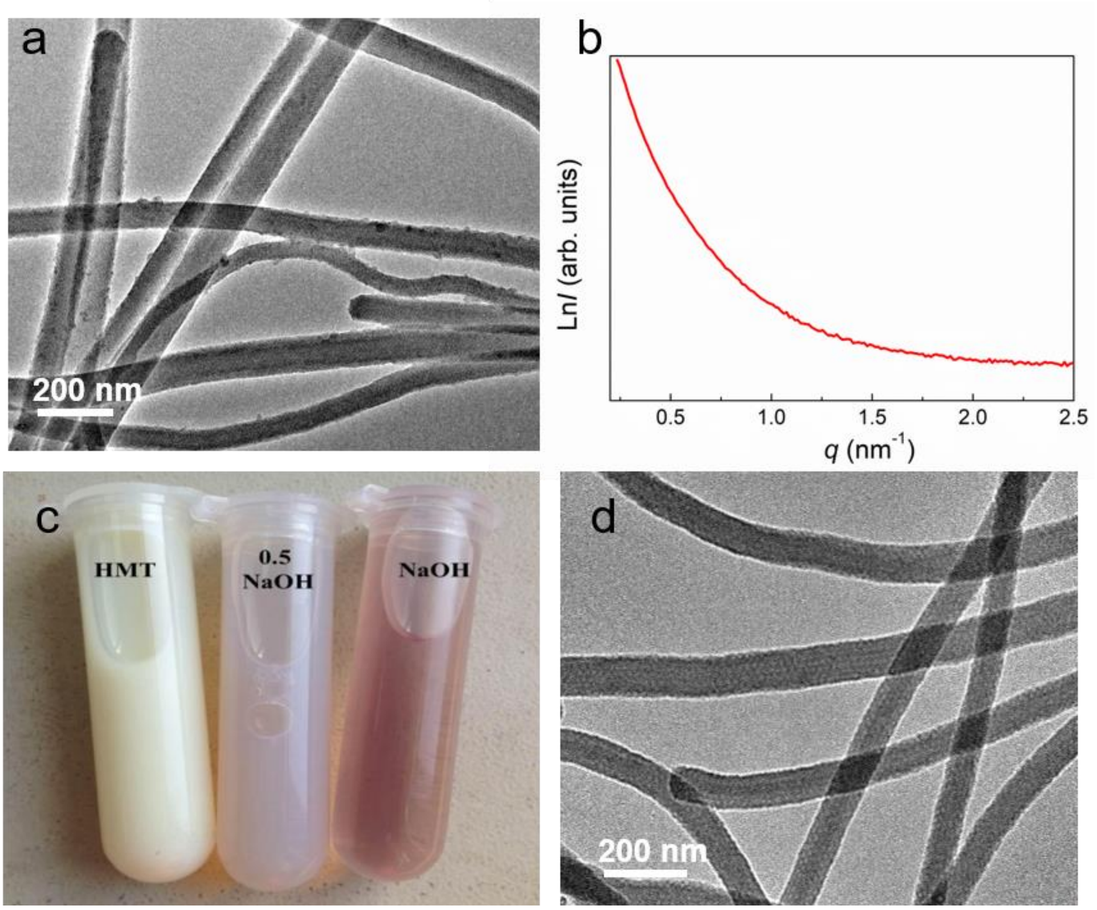

Supplementary Fig. 7. **Influence of catalyst on the pore structure of products.** (a) The TEM image and (b) SAXS pattern of the carbon nanofibers prepared by using $NH_3 \cdot H_2O$ as the catalyst. (c) Optical photographs of the reaction solutions using HMT, 50 % of NaOH, and NaOH as the catalysts. (d) The TEM image of the ordered mesoporous carbon nanofibers prepared by using HMT as the catalyst. The synthetic processes were performed similarly to the above Supplementary Method section except that the catalyst was varied. The polymeric samples were converted into carbon ones through a carbonization process at 800 °C for 2 h in N_2 atmosphere.

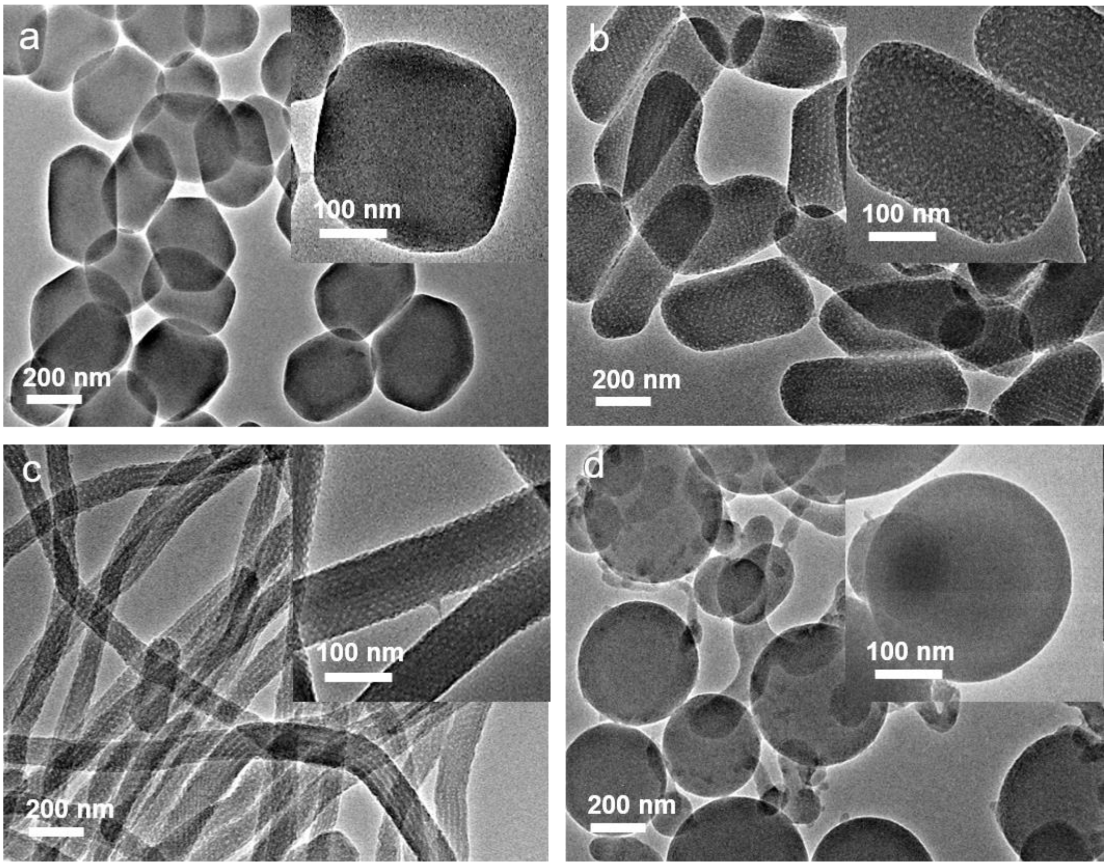

Supplementary Fig. 8. **Influence of HMT concentration on the morphology and pore structure of products.** The TEM images of the carbon materials prepared by the kinetically driven monomicelle oriented self-assembly approach using different HMT concentrations: (a) 0.2, (b) 0.4, (c) 0.8 and (d) 1.6 g L^{-1}. Insets are the corresponding magnification TEM images. The synthetic processes were carried out similarly to the above Supplementary Method section except that the HMT amount was varied. The polymeric samples were converted into carbon ones through a carbonization process at 800 °C for 2 h in N_2 atmosphere.

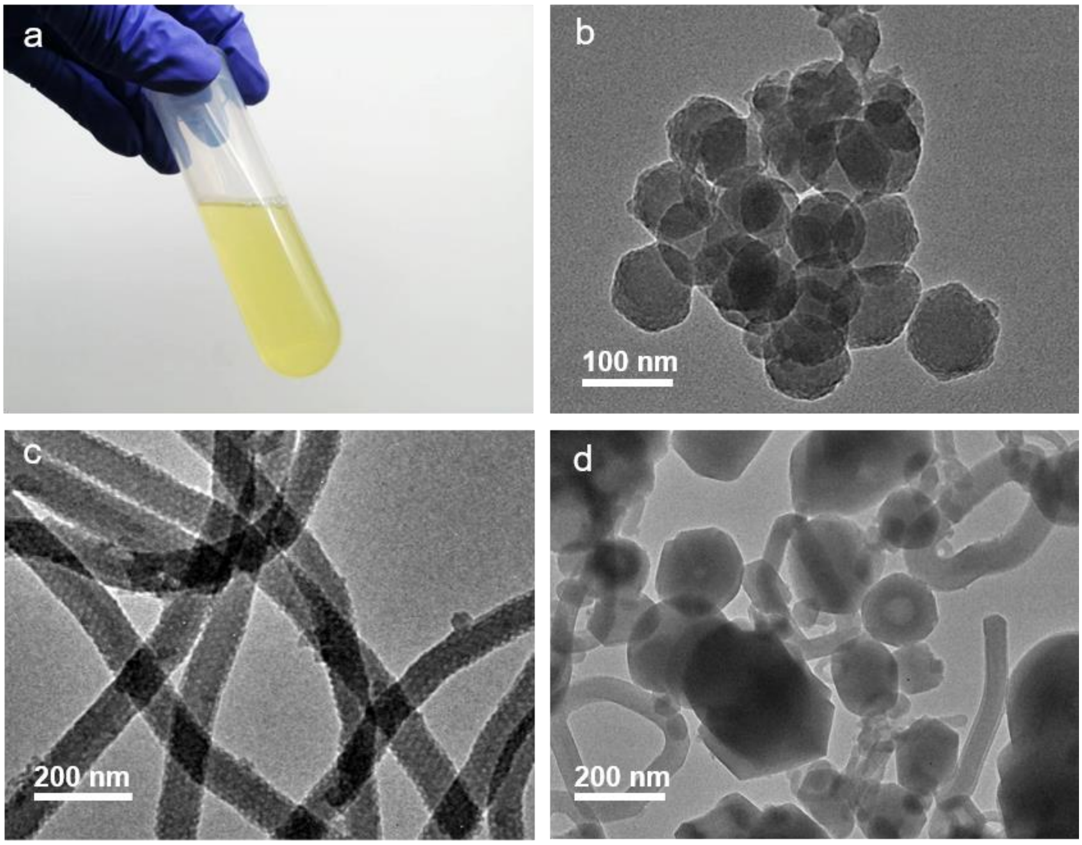

Supplementary Fig. 9. **Influence of reactant concentration on the morphology and pore structure of products.** The optical photograph and TEM images of the products prepared by the kinetically driven monomicelle oriented self-assembly approach using different reactant concentrations: (a) 50 %, (b) 75 %, (c) 100 % and (d) 125 %. Here, the reactant concentration used in the Method section is set to 100%. The synthetic processes were conducted similarly to the above Supplementary Method section except that the reactant concentration was varied. The polymeric samples were converted into carbon ones through a carbonization process at 800 °C for 2 h in N_2 atmosphere.

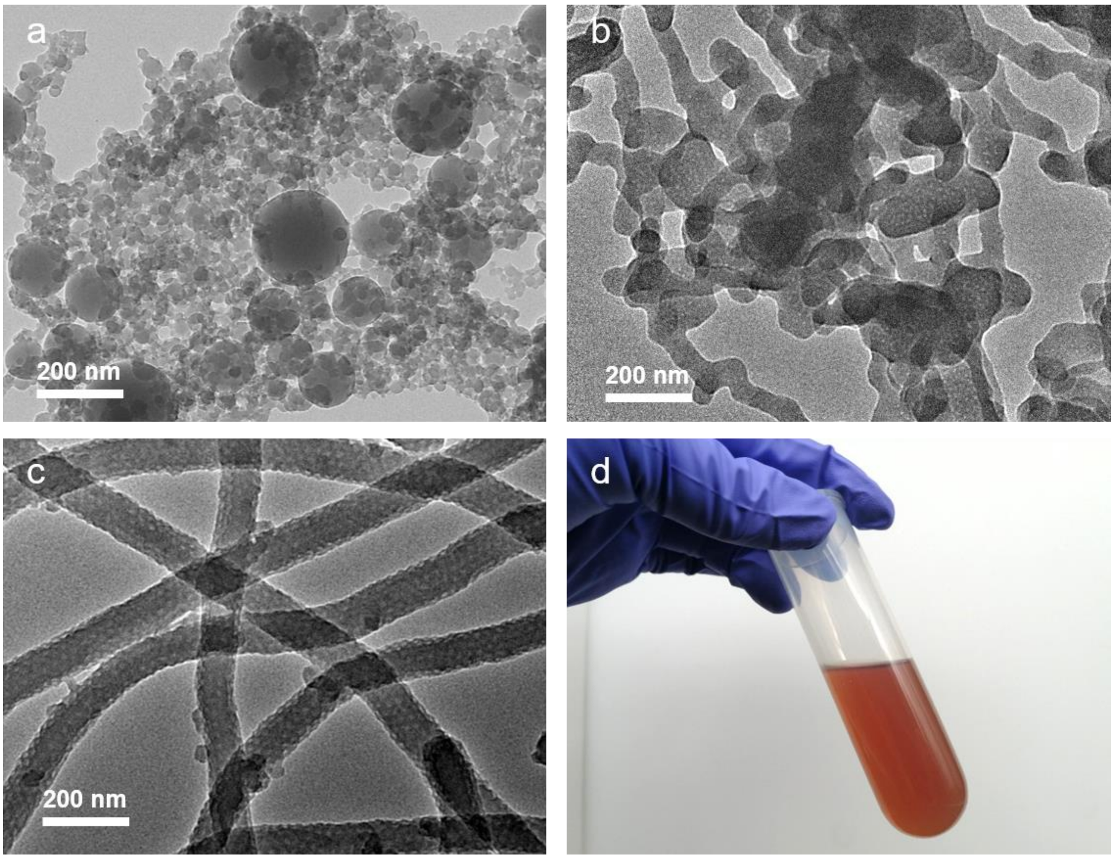

Supplementary Fig. 10. **Influence of pH value on the morphology and pore structure of products.** The TEM images and optical photograph of the products prepared by the kinetically driven monomicelle oriented self-assembly approach using different pH values: (a) ~5, (b) 8, (c) 9.5 and (d) 12. The pH of the reaction solution was adjusted by diluted hydrochloric acid (0.6 M) or potassium hydroxide (1.0 M) solutions. The synthetic processes were conducted similarly to the above Supplementary Method section except that the pH value was varied. The polymeric samples were converted into carbon ones through a carbonization process at 800 °C for 2 h in N_2 atmosphere.

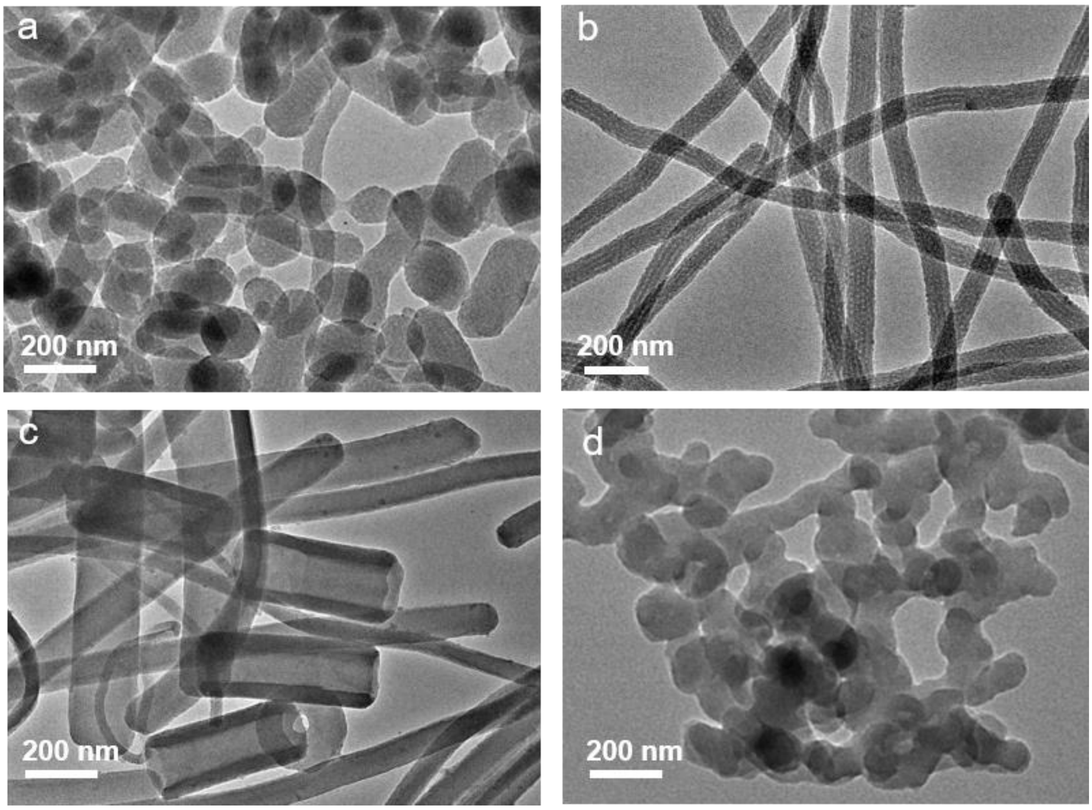

Supplementary Fig. 11. **Influence of reaction temperature on the morphology and pore structure of products**. The TEM images of the carbon materials prepared by the kinetically driven monomicelle oriented self-assembly approach at different reaction temperatures: (a) 80, (b) 100, (c) 120 and (d) 140 °C. The synthetic processes were performed similarly to the above Supplementary Method section except that the temperature was adjusted. The polymeric samples were converted into carbon ones through a carbonization process at 800 °C for 2 h in N_2 atmosphere.

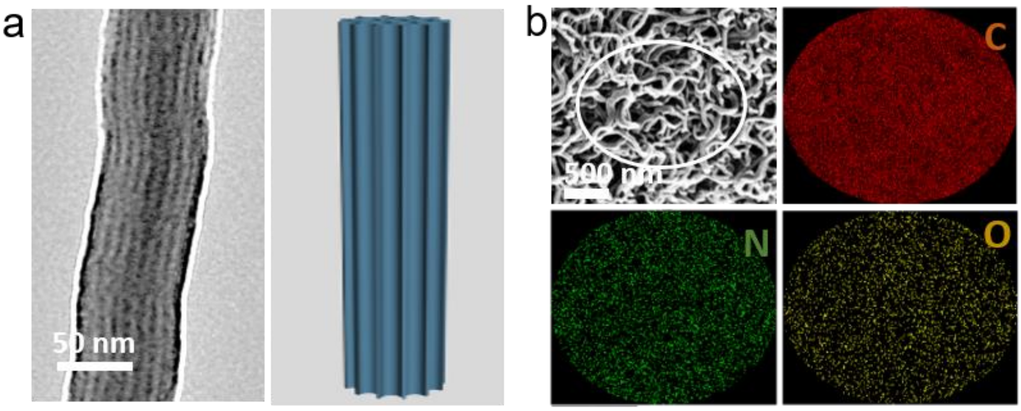

Supplementary Fig. 12. **Morphological and structural characterization of the ordered mesoporous carbon nanofibers**. (a) TEM image, structural model and (b) elemental mapping images of the hexagonal OMCFs prepared by the kinetically driven monomicelle oriented self-assembly approach. The polymeric samples were converted into carbon ones through a carbonization process at 800 °C for 2 h in N_2 atmosphere.

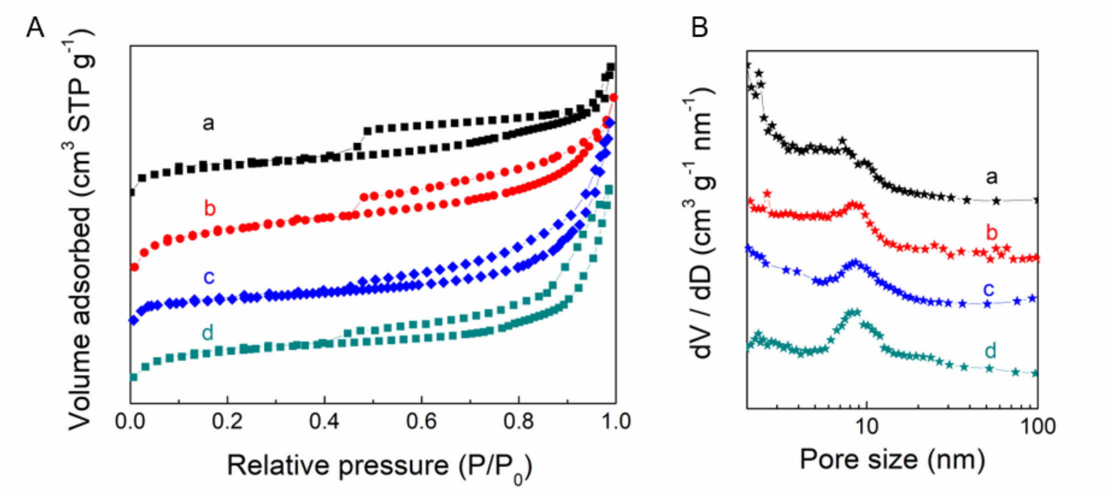

Supplementary Fig. 13. Pore characterization of the mesoporous carbon nanofibers. N_2 sorption isotherms (A) of the mesoporous carbon nanofibers prepared by the kinetically driven monomicelle oriented self-assembly approach using different F127/Resol mass ratios: (a) 0.30, (b) 0.35, (c) 0.40, and (d) 0.45. (B) The corresponding pore size distribution curves.

Supplementary Table 1. Physicochemical parameters of the resultant mesoporous carbon nanofibers prepared by the kinetically driven monomicelle oriented self-assembly approach using different F127/Resol mass ratios.

Samples	S_{BET}[a] [m^2 g^{-1}]	V_{total}[b] [cm^3 g^{-1}]	D_{pore}[c] [nm]
a	452	0.43	~6.0
b	421	0.49	6.8
c	347	0.42	7.4
d	264	0.47	8.0

[a] Specific surface areas (S_{BET}) were calculated using the adsorption branches in the range of P/P_0 = 0.05-0.25.

[b] Total pore volumes (V_{total}) were estimated based on the volume adsorbed at the P/P_0= ~ 0.995.

[c]Pore sizes (D_{pore}) were derived from the adsorption curves based on the BJH model.

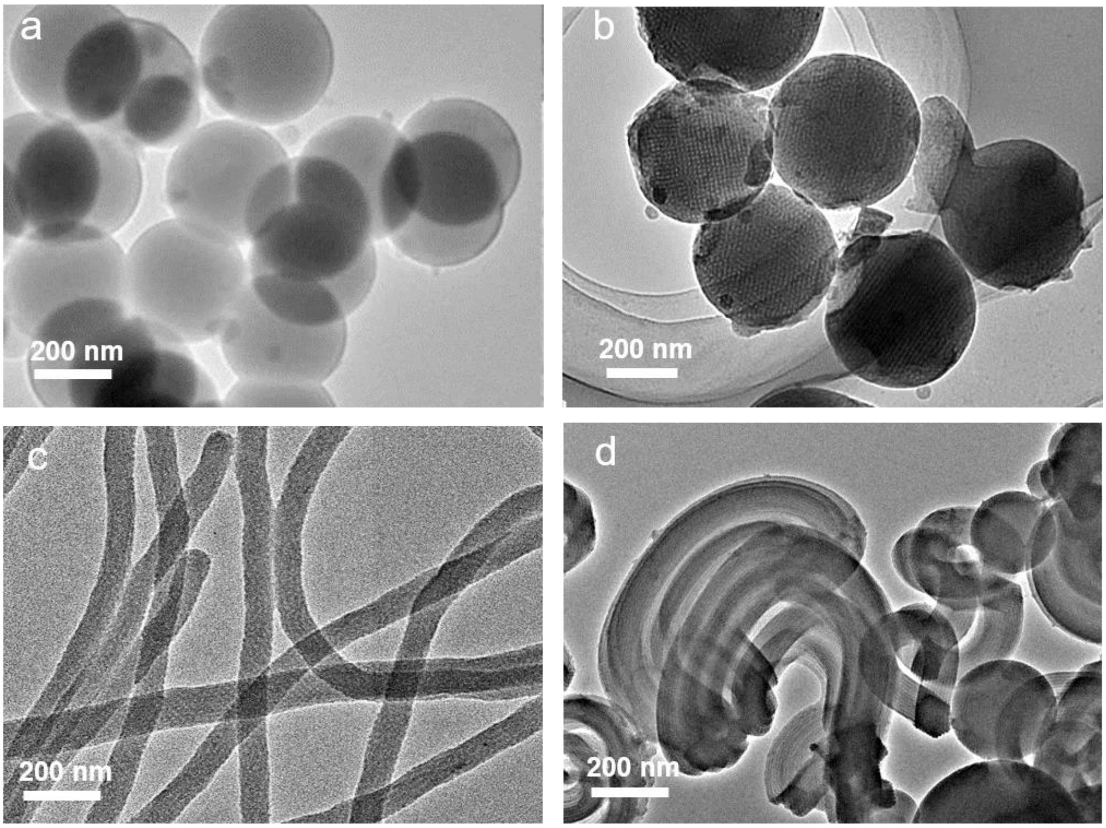

Supplementary Fig. 14. Influence of F127/Resol mass ratio on the morphology and pore structure of products. The TEM images of the carbon materials prepared by the kinetically driven monomicelle oriented self-assembly approach using different F127/Resol mass ratios: (a) 0, (b) 0.10, (c) 0.30 and (d) 0.60. The synthetic processes were conducted similarly to the above Supplementary Method section except that the F127/Resol mass ratio was varied. The polymeric samples were converted into carbon ones through a carbonization process at 800 °C for 2 h in N_2 atmosphere.

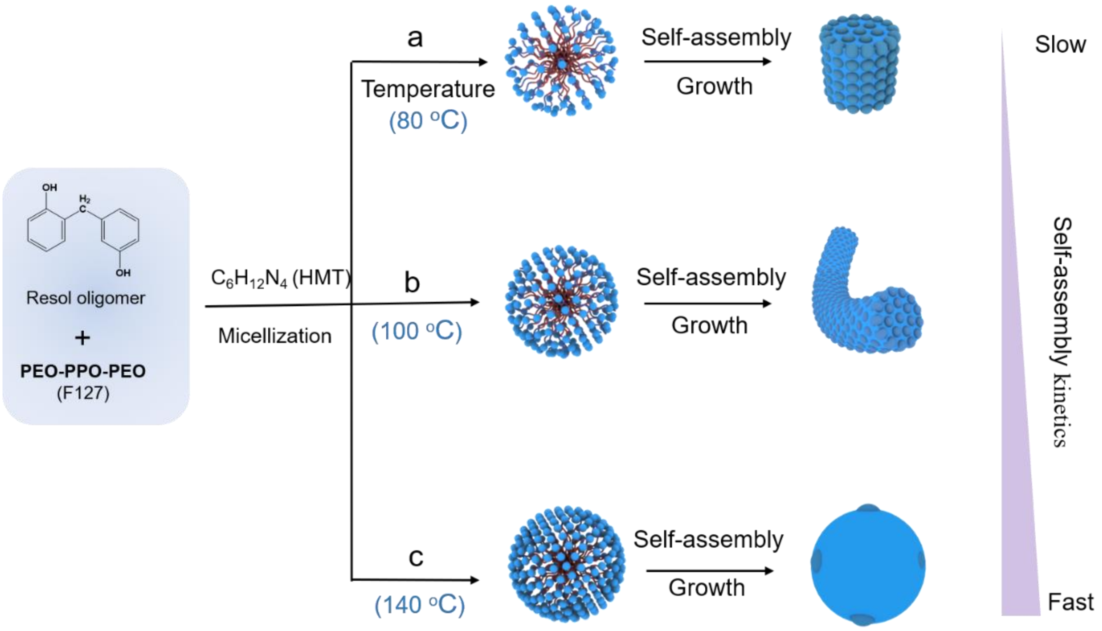

Supplementary Fig. 15. Schematic illustration of the mechanism of the kinetically driven monomicelle oriented self-assembly strategy. Three types of self-assembly modes were proposed when using different reaction temperatures: (a) 80, (b) 100, (c) 140 °C.

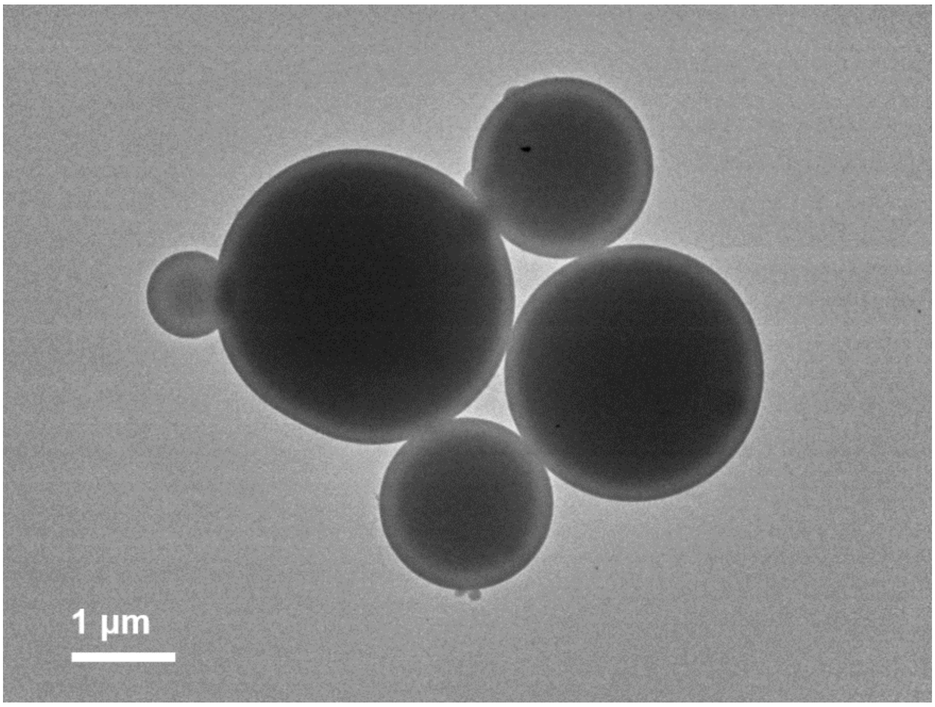

Supplementary Fig. 16. **Influence of reactant on the morphology and pore structure of products**. The TEM image of the product prepared by the kinetically driven monomicelle oriented self-assembly approach using pure formaldehyde as a curing agent. The polymeric sample was converted into carbon one through a carbonization process at 800 °C for 2 h in N_2 atmosphere.

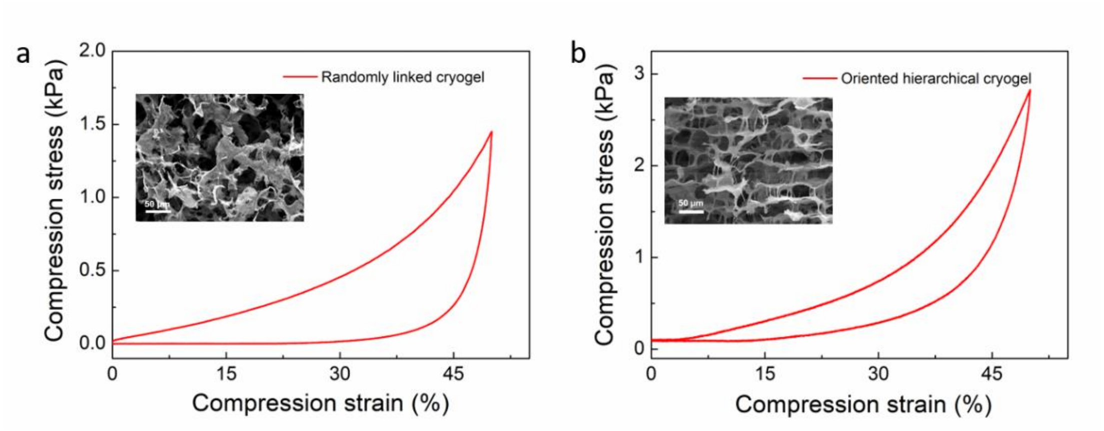

Supplementary Fig. 17. **Mechanical properties of the oriented hierarchical OMCFs cryogel and randomly linked nanofiber cryogel**. Stress-strain curves of (a) randomly linked and (b) oriented hierarchical OMCFs cryogels under a high strain compression, respectively. The inserted SEM images are of the corresponding samples.

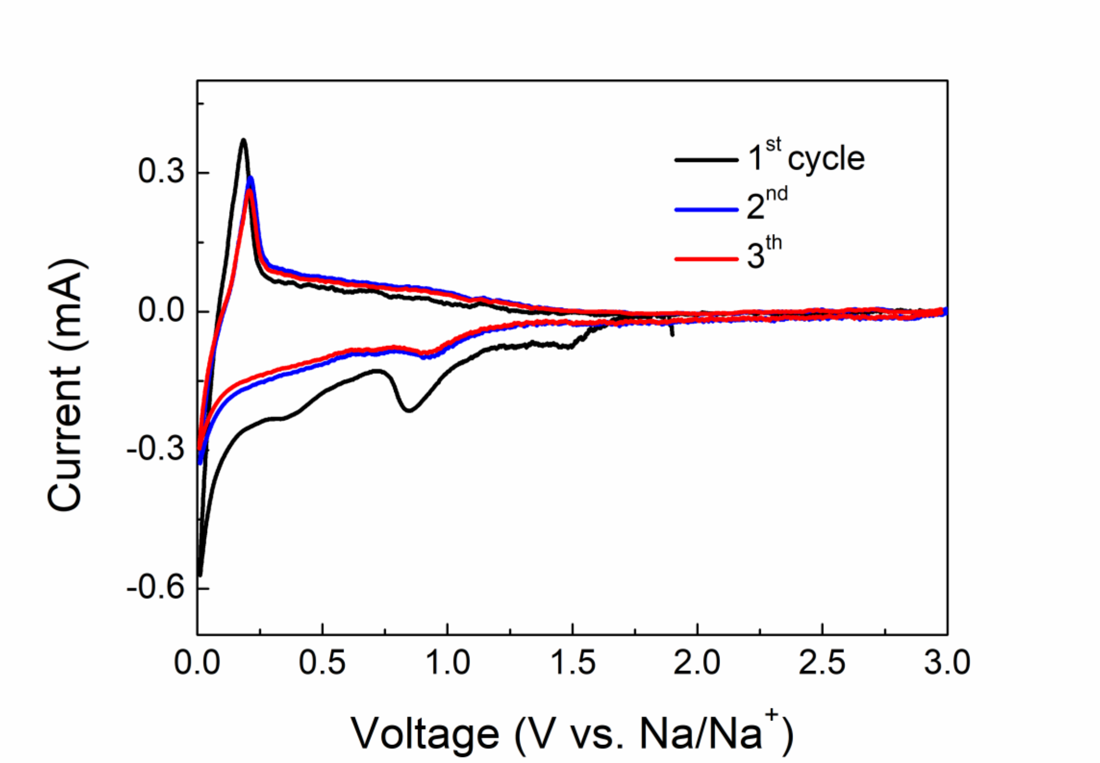

Supplementary Fig. 18. **Cyclic voltammograms of the ordered mesoporous carbon nanofibers electrode.** The cyclic voltammograms curves of the ordered mesoporous carbon nanofibers electrode at a scan rate of 0.2 mV s^{-1}.

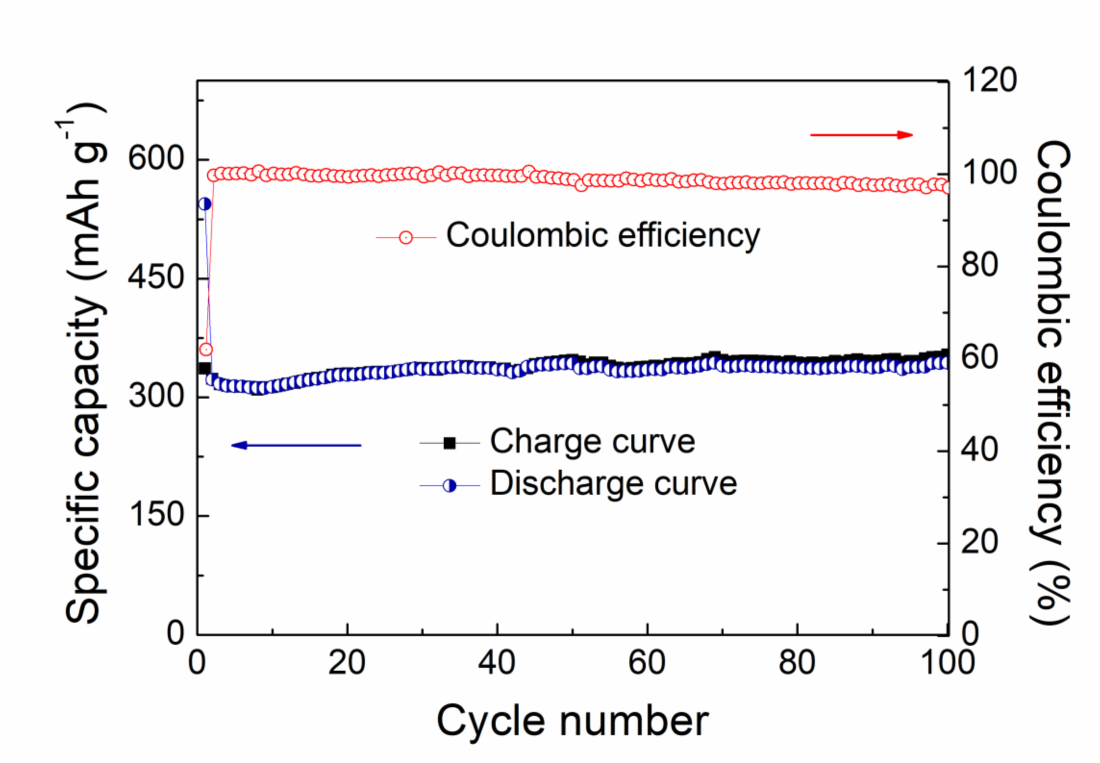

Supplementary Fig. 19. **Cyclic performance of the ordered mesoporous carbon nanofibers electrode.** The reversible tests of the ordered mesoporous carbon nanofibers electrode at a current density of 0.1 A g^{-1}.

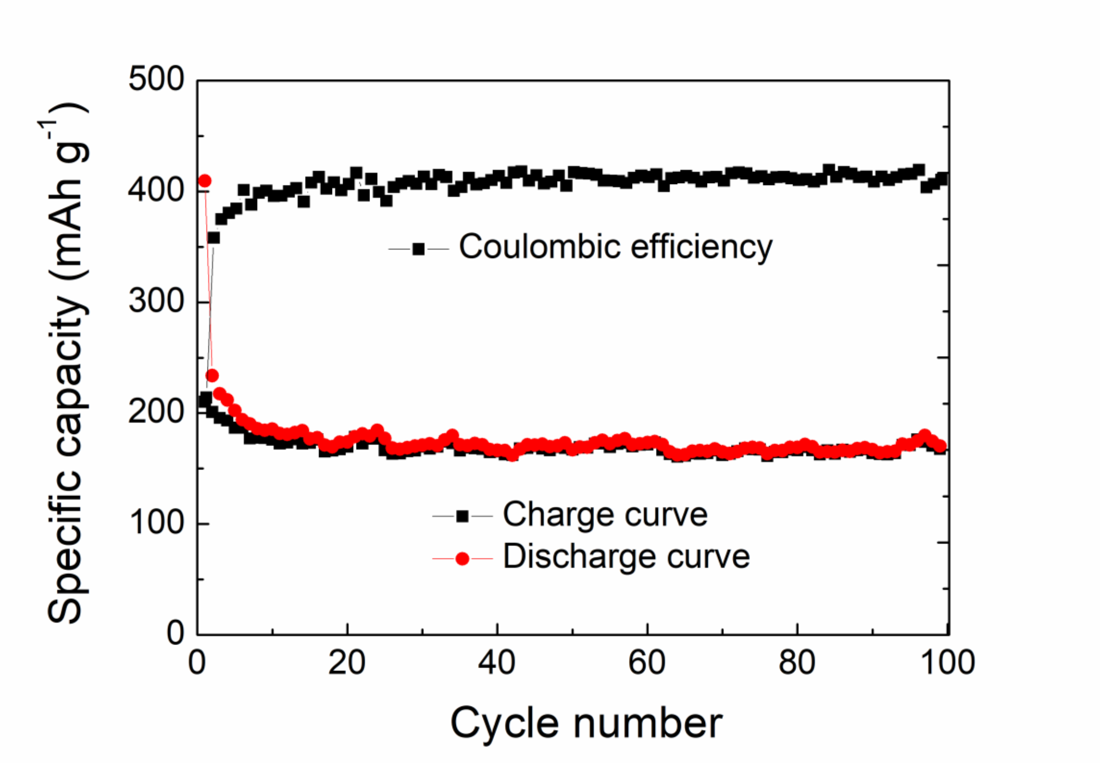

Supplementary Fig. 20. **Cyclic performance of the nonporous carbon particles electrode.** The reversible tests of the nonporous carbon particles at a current density of 0.1 A g^{-1}. The nonporous particles were synthesized by the kinetically driven monomicelle oriented self-assembly approach without adding of Pluronic F127 as a template.

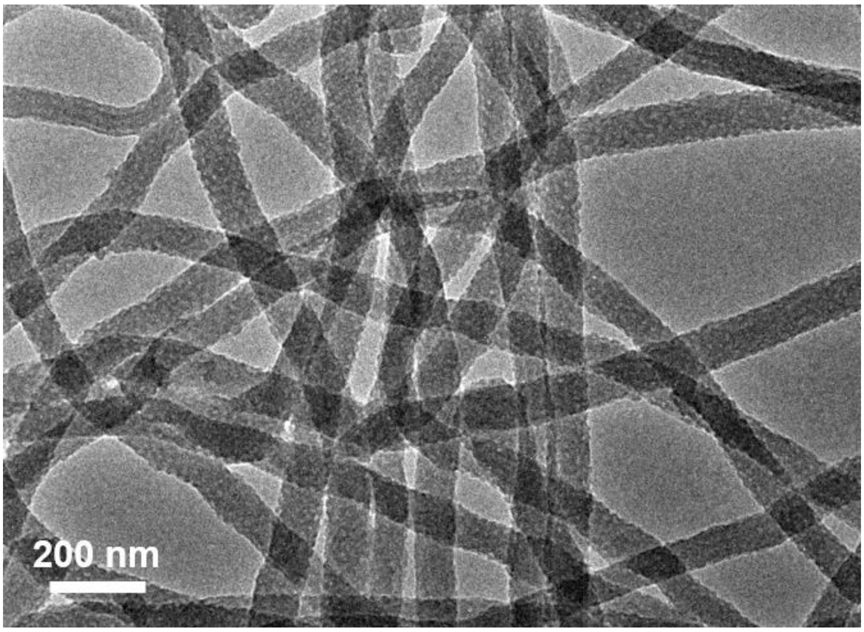

Supplementary Fig. 21. Stability of the ordered mesoporous carbon nanofibers. The TEM image of the ordered mesoporous carbon nanofibers after 100 cycles at a current density of 0.1 A g^{-1}.

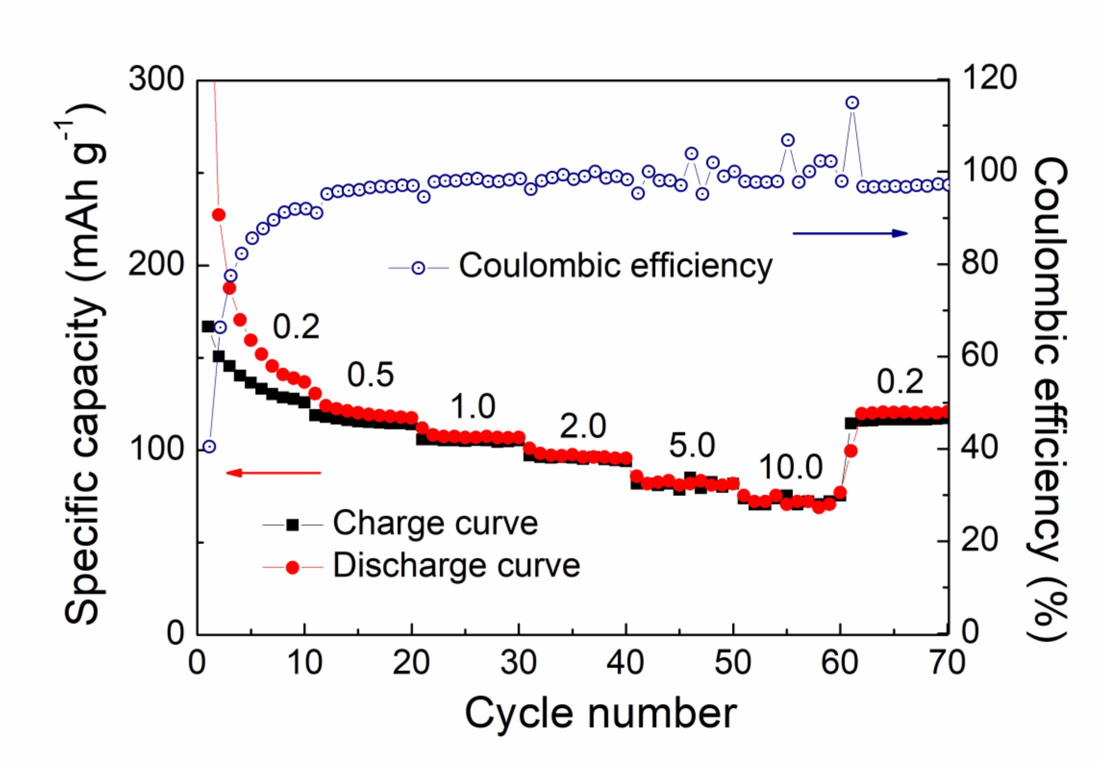

Supplementary Fig. 22. Rate capability of the nonporous carbon particles electrode. The current densities were increased from 0.2 to 10.0 A g^{-1}.

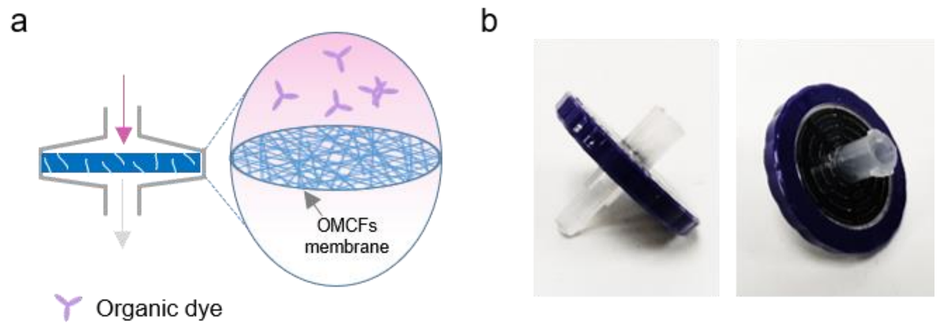

Supplementary Fig. 23. Design and characterization of the nanofilter device. (a) Schematic illustration and (b) optical photograph of the nanofilter device based on the ordered mesoporous carbon nanofibers cryogel.

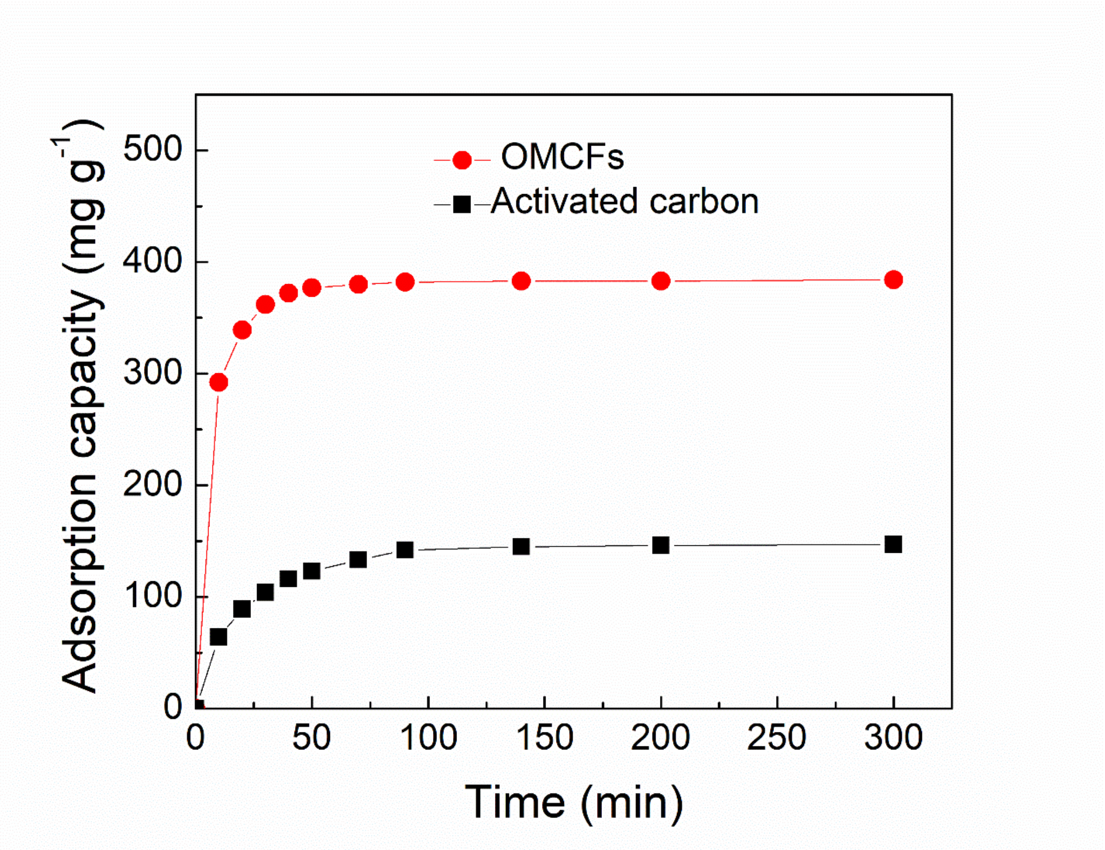

Supplementary Fig. 24. Adsorption performance of the ordered mesoporous carbon nanofibers and commercial activated carbon. Time-dependent adsorption capacities of Rhodamine B onto the ordered mesoporous carbon nanofibers and commercial activated carbon.

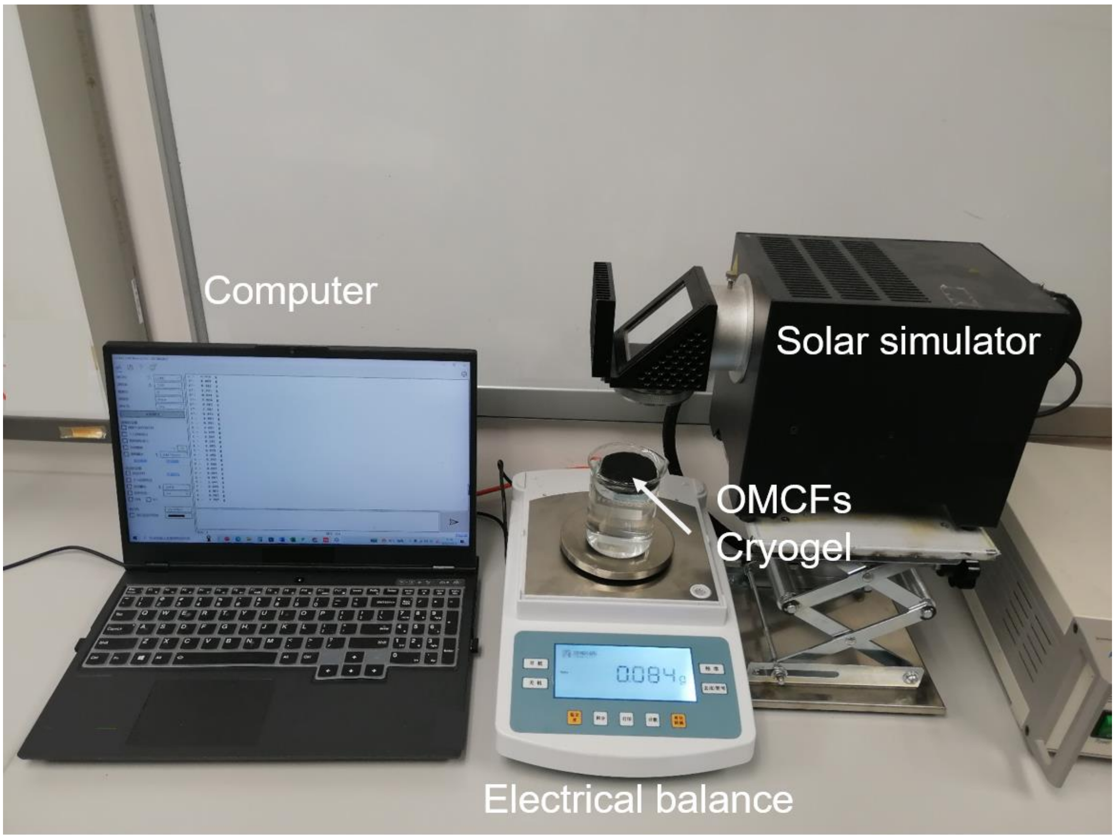

Supplementary Fig. 25. **Optical photograph of the solar water distillation system**. The system is based on the ordered mesoporous carbon nanofibers cryogel for seawater purification.

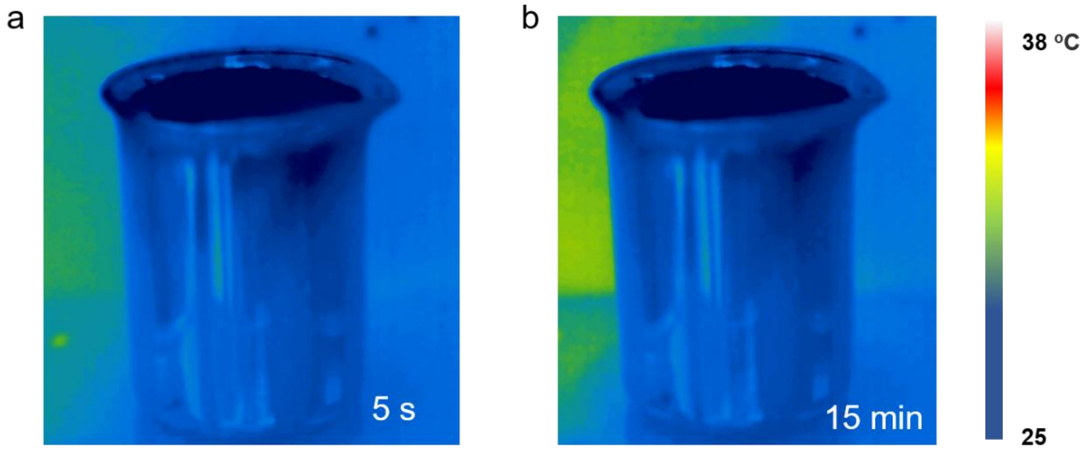

Supplementary Fig. 26. IR thermal images of the pure water under one-sun illumination.

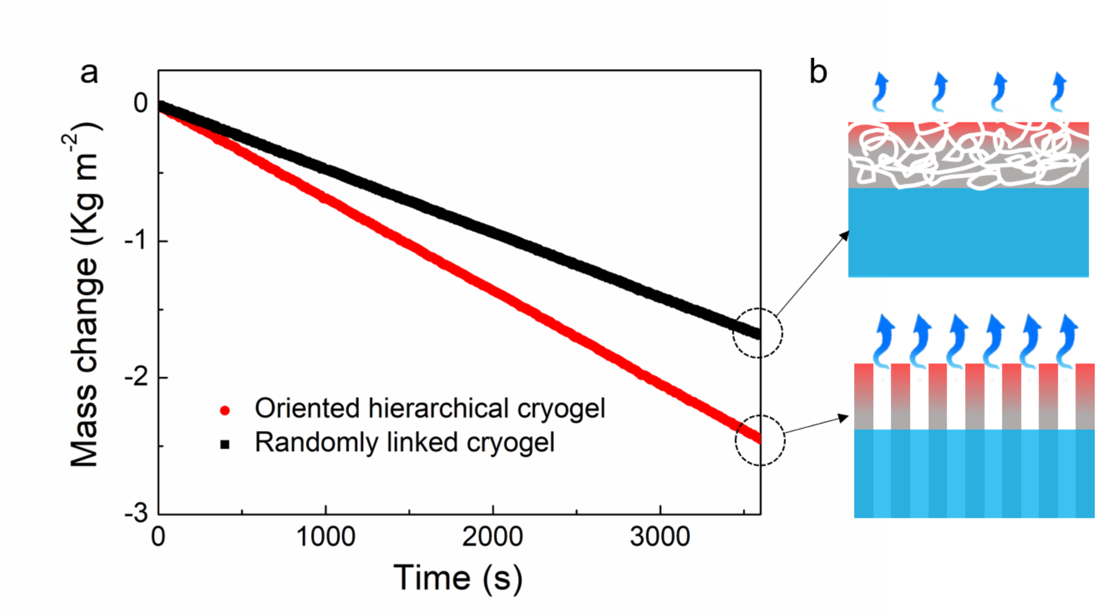

Supplementary Fig. 27. **Evaporation performance tests of the hierarchical OMCFs cryogel and randomly linked nanofiber cryogel**. (a) Mass change curves of the water on the hierarchical OMCFs cryogel and randomly linked nanofiber cryogel evaporators under one-sun illumination. (b) Schematic illustration showing the water transportation of the above-mentioned two solar-driven evaporators.

Supplementary Table 2. Comparison of the electrochemical performances of the ordered mesoporous carbon nanofiber electrode in this work with some carbon-based electrodes reported in the literature.

**Materials**	**Current density ($A\ g^{-1}$)**	**Cycle number**	**Specific capacity ($mAh\ g^{-1}$)**	**References**
Ultra-microporous carbon nanosheets	0.1	100	262	([3])
Micro-cuboid carbon	0.1	100	202	([4])
Interface optimized hard carbon	0.02	100	277	([5])
Nitrogen-rich hierarchically porous carbon	0.2	200	214	([6])
Sodium diphenyl ketone functioned carbon	0.5	100	238	([7])
1D micro-belts carbon paper	0.2	100	295	([8])
Defective hard carbon	0.2	100	187	([9])
Sulfur-doped hard carbon	0.1	100	320	([10])
Two-dimensional graphite	0.1	100	130	([11])
Nitrogen-doped hierarchical porous carbon	0.1	100	296	([12])
Nitrogen doped mesoporous carbon	0.1	100	234	([13])
**Ordered mesoporous carbon nanofibers （OMCFs）**	**0.1**	**100**	**346**	**This work**

Supplementary Table 3. Comparison of the water evaporation rate of the ordered mesoporous carbon nanofiber cryogels in this work with some carbon-based evaporators reported in the literature.

**Materials and structures**	**Evaporation rate ($kg\ m^{-2}\ h^{-1}$)**	**Solar intensity ($kW\ m^{-2}$)**	**References**
Self-supported carbon sponges	1.3	1	(14)
Carbon nanotubes film	1.4	1	(15)
Solar photovoltaic membrane	1.8	1	(16)
Organic-small-molecule photothermal materials	1.3	1	(17)
Bimodal porous structure with mesoporous carbon absorber	1.6	1	(18)
Carbon nanotubes resin based three-dimensional evaporator	2.6	1	(19)
Loofah sponge architectures with Cu nanoparticles in graphitic layers	1.5	1	(20)
Geopolymer-biomass mesoporous carbon composite device	1.6	1	(21)
Origami-inspired polymer foam	1.3	1	(22)
Nanocarbon cryogels	0.8	1	(23)
GO-based cryogels	1.7	2	(24)
Super-wicking black metal panel	1.2	1	(25)
**Ordered mesoporous carbon nanofibers （OMCFs）**	**2.4**	**1**	**This work**

3. Supplementary References

1. Meng Y*, et al.* Ordered Mesoporous Polymers and Homologous Carbon Frameworks: Amphiphilic Surfactant Templating and Direct Transformation. *Angew. Chem. Int. Ed.* **44**, 7053-7059 (2005).
2. Liu R*, et al.* Triconstituent co-assembly to ordered mesostructured polymer-silica and carbon-silica nanocomposites and large-pore mesoporous carbons with high surface areas. *J. Am. Chem. Soc.* **128**, 11652-11662 (2006).
3. Xia JL*, et al.* Hard carbon nanosheets with uniform ultramicropores and accessible functional groups showing high realistic capacity and superior rate performance for sodium-ion storage. *Adv. Mater.* **32**, 2000447 (2020).
4. Li Y, Kong M, Hu J, Zhou J. Carbon-microcuboid-supported phosphorus-coordinated single atomic copper with ultrahigh content and its abnormal modification to Na storage behaviors. *Adv. Energy Mater.* **10**, 2000400 (2020).
5. Tang Z*, et al.* Electrode-Electrolyte Interfacial Chemistry Modulation for Ultra-High Rate Sodium-Ion Batteries. *Angew. Chem. Int. Ed.* **61**, e202200475 (2022).
6. Hu X*, et al.* Nitrogen-rich hierarchically porous carbon as a high-rate anode material with ultra-stable cyclability and high capacity for capacitive sodium-ion batteries. *Nano Energy* **56**, 828-839 (2019).
7. Fang H*, et al.* Dual-Function Presodiation with Sodium Diphenyl Ketone towards Ultra-stable Hard Carbon Anodes for Sodium-Ion Batteries. *Angew. Chem. Int. Ed.* **62**, e202214717 (2023).
8. Hou B-H*, et al.* Self-Supporting, Flexible, Additive-Free, and Scalable Hard Carbon Paper Self-Interwoven by 1D Microbelts: Superb Room/Low-Temperature Sodium Storage and Working Mechanism. *Adv. Mater.* **31**, 1903125 (2019).
9. Li Z*, et al.* Defective Hard Carbon Anode for Na-Ion Batteries. *Chem. Mater.* **30**, 4536-4542 (2018).
10. Hong Z*, et al.* Rational Design and General Synthesis of S-Doped Hard Carbon with Tunable Doping Sites toward Excellent Na-Ion Storage Performance. *Adv. Mater.* **30**, 1802035 (2018).
11. Xu Z-L*, et al.* Tailoring sodium intercalation in graphite for high energy and power sodium ion batteries. *Nat. Commun.* **10**, 2598 (2019).
12. Zhou X*, et al.* Three-Dimensional Ordered Macroporous Metal-Organic Framework Single Crystal-Derived Nitrogen-Doped Hierarchical Porous Carbon for High-Performance Potassium-Ion Batteries. *Nano Lett.* **19**, 4965-4973 (2019).
13. Huang S*, et al.* Boosting Surface-Dominated Sodium Storage of Carbon Anode Enabled by Coupling Graphene Nanodomains, Nitrogen-Doping, and Nanoarchitecture Engineering. *Adv. Funct. Mater.* **32**, 2203279 (2022).
14. Zhu L*, et al.* Self-Contained Monolithic Carbon Sponges for Solar-Driven Interfacial Water Evaporation Distillation and Electricity Generation. *Adv. Energy Mater.* **8**, 1702149 (2018).
15. Xia Y*, et al.* Spatially isolating salt crystallisation from water evaporation for continuous solar steam generation and salt harvesting. *Energy Environ. Sci.* **12**, 1840-1847 (2019).
16. Wang W*, et al.* Simultaneous production of fresh water and electricity via multistage solar photovoltaic membrane distillation. *Nat. Commun.* **10**, 3012 (2019).

17. Chen G, *et al.* Biradical-Featured Stable Organic-Small-Molecule Photothermal Materials for Highly Efficient Solar-Driven Water Evaporation. *Adv. Mater.* **32**, 1908537 (2020).
18. Zhao H-Y, *et al.* Lotus-inspired evaporator with Janus wettability and bimodal pores for solar steam generation. *Cell Rep. Phys. Sci.* **1**, 100074 (2020).
19. Wu L, *et al.* Highly efficient three-dimensional solar evaporator for high salinity desalination by localized crystallization. *Nat. Commun.* **11**, 521 (2020).
20. Ren L, *et al.* Designing Carbonized Loofah Sponge Architectures with Plasmonic Cu Nanoparticles Encapsulated in Graphitic Layers for Highly Efficient Solar Vapor Generation. *Nano Lett.* **21**, 1709-1715 (2021).
21. Liu F, *et al.* Low cost, robust, environmentally friendly geopolymer-mesoporous carbon composites for efficient solar powered steam generation. *Adv. Funct. Mater.* **28**, 1803266 (2018).
22. Zhao L, *et al.* Shape-programmable interfacial solar evaporator with salt-precipitation monitoring function. *ACS Nano* **15**, 5752-5761 (2021).
23. Xia D, *et al.* Tuning the Electrical and Solar Thermal Heating Efficiencies of Nanocarbon Cryogels. *Chem. Mater.* **33**, 392-402 (2021).
24. Wang X, *et al.* An interfacial solar heating assisted liquid sorbent atmospheric water generator. *Angew. Chem. Int. Ed.* **131**, 12182-12186 (2019).
25. Singh SC, *et al.* Solar-trackable super-wicking black metal panel for photothermal water sanitation. *Nat. Sustain.* **3**, 938-946 (2020).
